# A novel anatomo-physiologic high-grade spondylolisthesis model to evaluate L5 nerve stretch injury after spondylolisthesis reduction

**DOI:** 10.1007/s10143-021-01721-z

**Published:** 2021-12-29

**Authors:** Basem Ishak, Clifford A. Pierre, Darius Ansari, Stefan Lachkar, Alexander von Glinski, Andreas W. Unterberg, Rod J. Oskouian, Jens R. Chapman

**Affiliations:** 1 Department of Neurosurgery, Heidelberg University Hospital, Im Neuenheimer Feld 400, 69120 Heidelberg, Germany; 2 Seattle Science Foundation, Seattle, WA USA; 3 Swedish Neuroscience Institute, Seattle, USA; 4 Department of Trauma Surgery, BG University Hospital Bochum, Bochum, Germany

**Keywords:** L5 nerve palsy, Stretch injury, Reduction, Spondylolisthesis, Cadaveric study

## Abstract

L5 nerve palsy is a well-known complication following reduction of high-grade spondylolisthesis. While several mechanisms for its occurrence have been proposed, the hypothesis of L5 nerve root strain or displacement secondary to mechanical reduction remains poorly studied. The aim of this cadaveric study is to determine changes in morphologic parameters of the L5 nerve root during simulated intraoperative reduction of high-grade spondylolisthesis. A standard posterior approach to the lumbosacral junction was performed in eight fresh-frozen cadavers with lumbosacral or lumbopelvic screw fixation. Wide decompressions of the spinal canal and L5 nerve roots with complete facetectomies were accomplished with full exposure of the L5 nerve roots. A 100% translational slip was provoked by release of the iliolumbar ligaments and cutting the disc with the attached anterior longitudinal ligament. To evaluate the path of the L5 nerves during reduction maneuvers, metal bars were inserted bilaterally at the inferomedial aspects of the L5 pedicle at a distance of 10 mm from the midpoint of the L5 pedicle screws. There was no measurable change in length of the L5 nerve roots after 50% and 100% reduction of spondylolisthesis. Mechanical strain or displacement during reduction is an unlikely cause of L5 nerve root injury. Further anatomical or physiological studies are necessary to explore alternative mechanisms of L5 nerve palsy in the setting of high-grade spondylolisthesis correction, and surgeons should favor extensive surgical decompression of the L5 nerve roots when feasible.

**Supplementary Information:**

The online version contains supplementary material available at 10.1007/s10143-021-01721-z.

## Introduction

Palsy of the fifth lumbar nerve is a well-known complication following surgical correction of high-grade spondylolisthesis due to traction on the nerve root during reduction [1, 10, 22]. The incidence of neurological deficits, such as nerve root, cauda equina, and peripheral nerve palsy, has been reported as high as 45%, although 90% of cases eventually improve upon follow-up [5, 10, 16, 21]. Besides traction injury to the L5 nerve root during the reduction process, other possible mechanisms of L5 palsy include neurovascular dysfunction, foraminal morphometry, temporary displacement of the L5 nerve root during decompression, and hyperextension of the patient during positioning [4, 9, 11, 19, 21]. In order to minimize postoperative L5 nerve palsy after reduction of high-grade spondylolisthesis, several authors hypothesize that most of the total L5 nerve strain occurs during the second half of reduction, and therefore suggest a reduction of no more than 50% and/or decompression of the L5 nerve roots prior to reduction [10, 13, 20]. Other potentially protective measures include electromyography (EMG) monitoring and postoperative positioning of the patient in a hip- and knee-flexed position with progressive straightening leg extension over several days [9, 19, 20].

Aside from few case reports, which are predominantly limited to neurological deficits in general, the incidence of L5 nerve palsy in spinal surgery has not been well described in the literature. Although postoperative L5 nerve palsy with foot drop and dysesthesia is not life-threatening, they frequently necessitate longer hospitalization, intensive physiotherapy, and rehabilitation and can lead to permanent disability in the absence of spontaneous recovery [17]. Furthermore, L5 nerve palsies in spinal surgery impair patients’ ability to perform activities of daily living and burden the healthcare system with higher treatment costs [17].

Presently, the true mechanism underlying postoperative L5 nerve palsy remains unclear and is likely multifactorial in origin. However, a deeper understanding of the morphological parameters and anatomic relationships of the L5 nerve root during specific surgical procedures could add further insight to the stretch injury hypothesis. Herein, we simulate intraoperative reduction maneuvers for high-grade spondylolisthesis in order to analyze the anatomic displacement and stretching of the L5 nerve root and evaluate this process as a potential cause for postoperative L5 nerve palsy.

## Methods

**Fig. 1:**
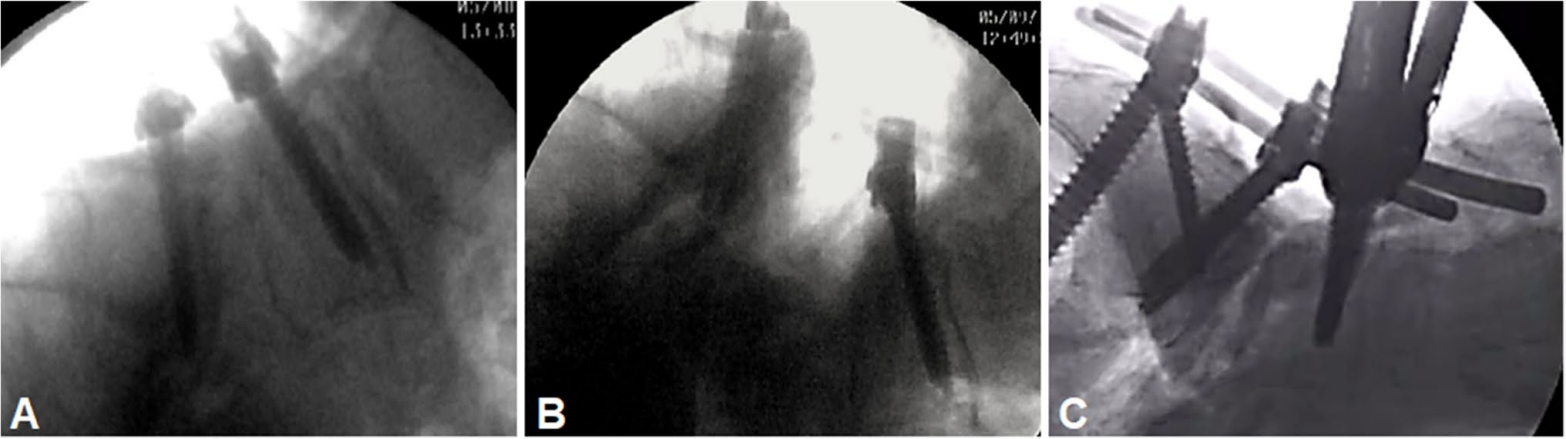
**A** Standard posterior approach to the lumbosacral junction with screw placement. **B** A 100% slip was provoked at the lumbosacral junction. **C** Reposition was obtained through lumbosacral or lumbopelvic fixation

**Fig. 2:**
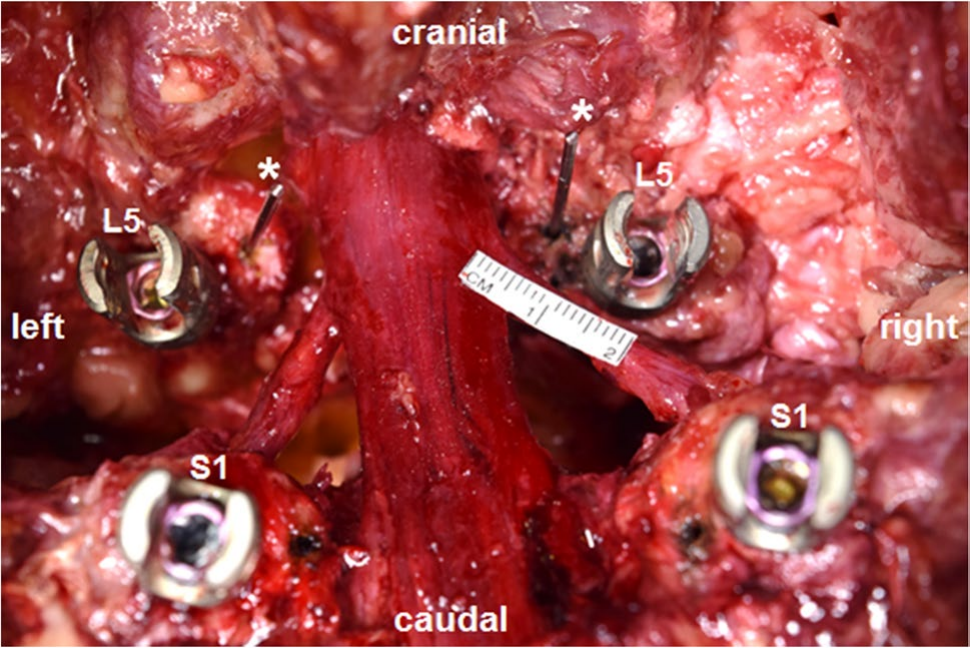
Two metal bars (asterisks) were inserted at the medial inferior aspect of the L5 pedicle, bilaterally at a distance of 10 mm to the midpoint of the L5 pedicle screw. A ruler was then placed on the L5 nerve root to measure movement after 50% and 100% reduction

In this novel anatomo-physiologic high-grade spondylolisthesis model, we attempted to mimic ideal physiological conditions with intact muscular and neural structures. An institutional review board approval was not required because the dissections were performed on deidentified cadaveric specimens. Sixteen sides of eight fresh-frozen Caucasian (*n* = 7) and African-American (*n* = 1) cadaveric specimens were examined (Table 1). The specimens were derived from 6 males and 2 females, with a mean age at death of 78.4 years (range 65–89 years). We performed a standard posterior approach to the lumbosacral junction with lumbosacral or lumbopelvic fixation (Globus Medical, Audubon, PA, USA). Wide decompressions of the spinal canal and L5 nerve roots with complete facetectomies were carried out. The lumbar vertebral bodies and the L5 nerve roots on both sides were then exposed with meticulous dissection to avoid inadvertent structural neural injury. The intervertebral disc with the attaching anterior longitudinal ligament was removed. To provoke a 100% slip, the iliolumbar ligaments were divided, and a hard box was placed beneath the lower lumbar spine as a pivotal point (Fig. 1A, B). Movement of the L5 nerve root was then tested after 50% and 100% reduction using 5.5-mm titanium rods (Globus Medical, Audubon, PA, USA) (Fig. 1C). To evaluate the path of the L5 nerves during reduction maneuvers, a metal bar was inserted at the inferomedial aspect of the L5 pedicle, bilaterally at a distance of 10 mm to the midpoint of the L5 pedicle screw based on a previously described protocol by Ebraheim et al. [6] (Fig. 2). Thereafter, a ruler was placed on the L5 nerve root to measure movement after 50% and 100% reduction (Fig. 2), and strain on each nerve root was assessed using a nerve hook.

**Table 1:** Characteristics of the tested specimens

Specimen no	Sex	Age
1	M	74
2	M	87
3	F	89
4	M	81
5	M	85
6	M	65
7	F	79
8	M	67

All observations were performed by two senior researchers from the author board. Movement was considered as measurable when L5 nerve excursion of at least 1 mm occurred. Statistical differences between gender and sides were compared and evaluated using a chi-squared test. A *p*-value of < 0.05 was determined a priori to represent statistical significance. SPSS 24 (SPSS, Munich, Germany) was used for statistical analysis. As this was a cadaveric study, Institutional Review Board approval was not necessary nor sought.

## Results

**Fig. 3:**
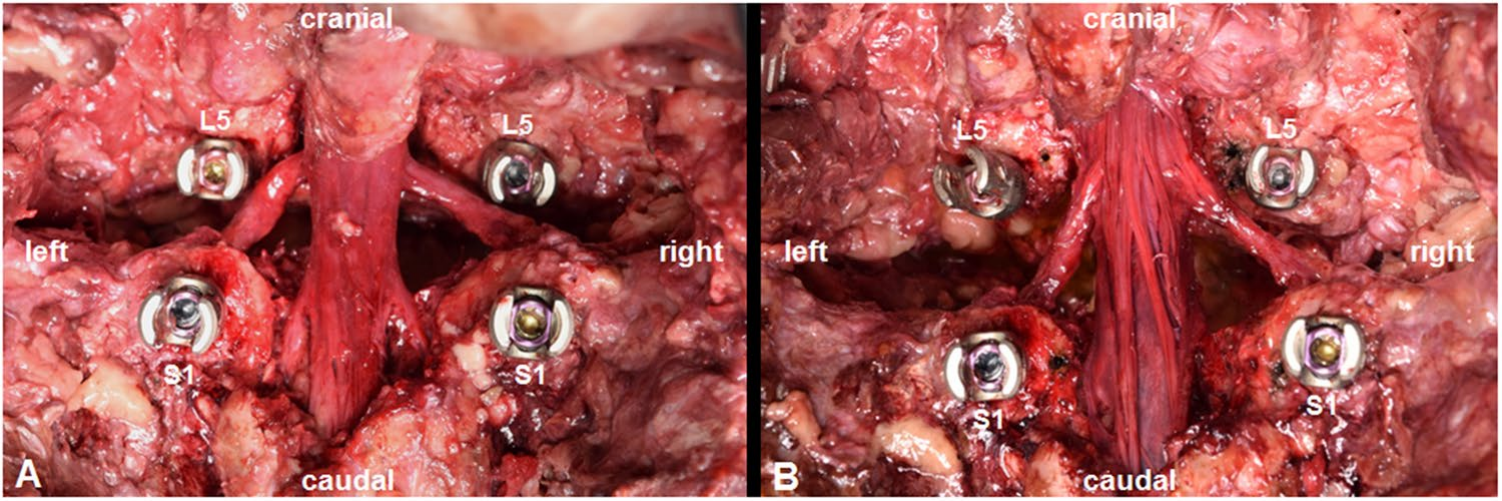
Tactile assessment of L5 nerve strain after 100% slip (**A**). Opening the dura to determine intradural strain on rootlets (**B**)

No signs of L5 nerve root compression were found in any of the 16 sides studied. There was no significant difference in terms of gender and sides (*p* < 0.05). In this spondylolisthesis model with a 100% slip, reduction of 50% did not result in any measurable movement bilaterally (< 1 mm). Similarly, during 100% reduction, movement of the L5 nerve was also unmeasurable bilaterally (Video 1, Supplemental Material). Strain measurement using a nerve hook did not reveal tension on any of the studied nerve roots (Fig. 3A; Video 2, Supplemental Material). Afterwards, the dura mater was opened to exclude any intradural strain (Fig. 3B) after a 100% slip.

## Discussion

Several mechanisms have been proposed as etiologies of isolated postoperative L5 nerve root deficits. Among these include traction during the reduction process of high-grade spondylolisthesis, direct trauma during nerve root decompression, or instrumentation of the L5 vertebral body [9]. A delayed postoperative L5 nerve palsy of vascular origin resulting in endoneurial hypoxia has also been described [19].

Although there is little agreement on the precipitating factors for L5 nerve palsy specifically after reduction of high-grade spondylolisthesis, prior studies have shown that most of the L5 nerve strain occurs in the second half of high-grade spondylolisthesis reduction [7, 20]. On the one hand, transforaminal ligaments in the lumbar intervertebral foramen, such as the superior and inferior costotransverse ligaments, which are mostly distributed in the L5-S1 neuroforamen, may influence the L5 nerve root during the reduction process [24]. On the other hand, extra-foraminal motion of the L5 nerve root during patient positioning could also contribute to a stretch injury of the L5 nerve root due to nerve excursion and displacement of intrathecal nerve roots [2, 12, 18]. Osteophytes arising from the lower L5 and upper S1 may also contribute to the development of postoperative L5 palsy [14]. In a recently published anatomical study, L5 nerve movement and displacement during flexion/extension of the hip and lower lumbar spine have been quantified [8]. The authors did not find any discernable movement of the L5 nerve root or its roots after flexion and extension of the hip and lower lumbar spine.

To this end, the goal of this cadaveric study was to determine movement of the L5 nerve root bilaterally after reduction maneuvers for high-grade spondylolisthesis in order to analyze the anatomic displacement and stretching of the L5 nerve roots.

In 1996, Petraco et al. quantified changes in the length of the L5 nerve root during reduction in a semi-anatomical high-grade spondylolisthesis model [20]. In four cadaveric specimens, the L5 and S1 vertebral body were cleaned thoroughly from soft tissue, including all ligaments and neural structures. A pars defect was created by removing the posterior bony elements. Following fixation of the S1 vertebral body onto a customized frame, the L5 vertebral body was mounted above, permitting movement in all directions. An adjustable jig was placed between these two bodies to allow 100% slip. The L5 nerve roots were replaced by inelastic cords and two reference points were chosen to determine changes in length of the L5 nerve root after reduction of a high-grade spondylolisthesis. In concordance with the previous literature at that time, the authors concluded that the first 50% of reduction of a high-grade spondylolisthesis with 100% slippage resulted in 4% strain of the L5 nerve which is unlikely to cause a nerve injury. In contrast to this, a complete (100%) reduction caused 14% strain, which bears a high possibility of causing an L5 nerve injury [20].

The primary limitation of the conclusions by Petraco et al. is that they were drawn from a non-physiologic anatomical model, specifically, one in which the natural neural elements and ligaments were simulated using a non-organic inelastic cord. Nevertheless, it remains the first and only study aimed at elucidating the role of stretch injury in the genesis of L5 nerve palsy after reduction of high-grade spondylolisthesis.

Despite this previous study by Petraco et. al. [20], uncertainty remains regarding the threshold of nerve strain that can occur prior to irreversible nerve injury. In order to quantify measurable nerve injury stretching, Wall et al. [23] developed an animal model in which the tibial nerve of 24 rabbits was stretched by 0%, 6%, or 12% of its length with maintaining the strain for 1 h. Among their findings were that a 6% strain led to 70% decrease of the amplitude of the action potential for 1 h, but recovered fully thereafter, while 12% strain caused irreversible damage to the nerve [23]. In a similar model, Brown et al. [3] examined the electrophysiologic properties of the tibial nerve in 30 rabbits by stretching the nerve for 2 h by 0%, 8%, and 15% of its original length. The action potential did not significantly decrease after 8% strain, but a 15% strain produced a 99% drop of amplitude in the action potential [3]. Lundborg et al. studied the microcirculation in rabbit tibial nerves [15]. Elongation of 8% could be tolerated without impairment of intraneural blood flow, compared to 15% strain, which led to interruption of blood flow and consequent complete ischemia, indicating permanent nerve injury [15]. The results of these experimental studies appear to suggest a strain threshold exists whereafter irreversible nerve injury occurs and is highly dependent upon the force and elongation time that the nerve is subjected to tension [15].

In the present anatomical study, we evaluated movement of the L5 nerve root during different reduction maneuvers and, importantly, demonstrated no significant movement or displacement of the L5 nerve root during its intra- and extra-foraminal course. Our findings suggest that anatomical attachments, such as the dura and intra-foraminal ligaments, do not contribute to stretching of the L5 nerve and are unlikely sources of intraoperative injury.

### Disadvantages of cadaveric dissections

Compared to in vivo studies, there have been published reports that adults with high-grade spondylolisthesis remain minimally symptomatic although the progression of their slip is more commonly due to degenerative changes from the aging spine that can produce scar formation around the L5 nerve roots [19]. This can cause tissue shrinkage or stiffening of the nerve roots. Furthermore, preexisting periforaminal spondylitic osteophyte formation may also contribute to L5 nerve palsy which is not given when an instant slip is created [5]. While our model did not demonstrate evidence of L5 stretch injury during reduction maneuvers, from a clinical scenario perspective, some anatomical causes of L5 nerve injury may involve inadequate neuroforaminal decompression due to bony overgrowth and ligament hypertrophy or transient anterior displacement of the L5 nerves during surgical exposure [21]. Direct trauma-related root injury due to manipulation can also not be quantified in cadavers. Extensive soft tissue dissection and intraoperative blood loss are other known precipitating factors that could lead to the reported endoneurial hypoxia [18] which can also not be addressed in a cadaveric study, although we did not appreciate significant displacement of the regional vasculature during the reduction maneuvers. Another disadvantage in cadaveric specimens is the fact that nerve strain cannot be quantified properly by using electrophysiology.

### Limitations

The major limitation of the present study is the restriction of the analysis to translational slip only, rather than evaluation of different slip angles, disc angles, disc heights, or kyphotic influence. Manipulation of these parameters, although certainly of anatomical relevance, would require more extensive dissection, which may not be representative of a common posterior exposure of the lumbosacral junction and may therefore restrict the generalizability of the findings for surgical applications. For similar reasons, connective tissue outside of the surgical field was not evaluated due to the aim of modeling a physiological surgical scenario. Notably, although the psoas major muscle may have some influence on the course of the L5 nerve, we believe the intraoperative relevance would be minimal as the patient is likely to be anesthetized and therefore paralyzed for the duration of the procedure. Finally, as with all cadaveric studies, differences between the properties of cadaveric and living tissue must be considered in the interpretation of the present results.

## Conclusion

L5 nerve palsy is likely to be multifactorial and the underlying pathophysiology remains unclear. In this anatomical model, we were unable to demonstrate significant displacement of the L5 nerve root following 50% and 100% reduction of spondylolisthesis with 100% slip. These findings suggest that traction injury during spondylolisthesis reduction is unlikely to be a significant contributor to postoperative L5 palsy. However, extensive decompression of the L5 nerve roots, as performed in the present study, may help to reduce incidence of L5 nerve palsy.

## Supplementary Information

Below is the link to the electronic supplementary material.

Supplementary file1 (MOV 20558 KB)

Supplementary file2 (MOV 22820 KB)

## Data Availability

Data are available upon request.
